# Health-related quality of life, anxiety and depression in the diagnostic phase of suspected cancer, and the influence of diagnosis

**DOI:** 10.1186/s12955-016-0484-9

**Published:** 2016-05-20

**Authors:** Ellen Moseholm, Susan Rydahl-Hansen, Dorthe Overgaard, Hanne S. Wengel, Rikke Frederiksen, Malene Brandt, Bjarne Ø. Lindhardt

**Affiliations:** Department of Pulmonary and Infectious Diseases, University Hospital of Copenhagen, Nordsjælland, Dyrehavevej 29, DK - 3400 Hillerød, Denmark; Research Unit of Clinical Nursing, Bispebjerg and Frederiksberg University Hospital, and Department of Public Health, Section for Nursing, Aarhus University, Bispebjerg Bakke 23, 20D, Copenhagen, NV DK- 2400 Denmark; Department of Nursing, Metropolitan University College, Tagensvej 86, Copenhagen, N DK - 2200 Denmark; Department of Endocrinology, Copenhagen University Hospital, Herlev, Herlev Ringvej 75, Building 64, Herlev, DK-2730 Denmark; Department of Gastroenterology, Bispebjerg and Frederiksberg University Hospital, Bispebjerg Bakke 23, 60, Copenhagen, NV DK- 2400 Denmark; Department of Infectious Diseases, Copenhagen University Hospital, Hvidovre, Kettegård Alle 30, Department 144, Hvidovre, DK- 2650 Denmark

**Keywords:** Cancer, Oncology, Pre-diagnosis, Health-related quality of life, Anxiety, Depression

## Abstract

**Background:**

Undergoing diagnostic evaluation for cancer has been associated with a high prevalence of anxiety and depression and affected health-related quality of life (HRQoL). The aims of this study were to assess HRQoL, anxiety, and depression pre- and post-diagnosis in patients undergoing diagnostic evaluations for cancer due to non-specific symptoms; to examine changes over time in relation to final diagnosis (cancer yes/no); and to assess the predictive value of pre-diagnostic psychological, socio-demographic and clinical factors.

**Methods:**

A prospective, multicenter survey study of patients suspected to have cancer based on non-specific symptoms was performed. Participants completed the EORTC-QLQ-C30 quality of life scale, HADS, SOC-13 and self-rated health before and after completing diagnostic evaluations. Intra- and inter-group differences between patients diagnosed with cancer versus patients with non-cancer diagnoses were calculated. The impact of baseline psychological, socio-demographic, and medical factors on HRQoL, anxiety and depression at follow-up was explored by bootstrapped multivariate linear regression analyses and logistic regression analyses.

**Results:**

A total of 838 patients participated in this study; 679 (81 %) completed the follow-up. Twenty-two percent of the patients received a cancer diagnosis at the end of the follow-up. Patients presented initially with a high burden of symptoms and affected role and emotional functioning and global health/QL, irrespective of diagnosis. The prevalence of clinical anxiety prior to knowledge of the diagnosis was 32 % in patients with cancer and 35 % in patients who received a non-cancer diagnosis. HRQoL and anxiety improved after diagnosis, and a larger improvement was seen in patients who received a non-cancer diagnosis. There were no intra- or inter-group differences in the depression scores. The strongest predictors of global QL, anxiety, and depression after a known diagnosis were baseline scores, co-morbidity and poor self-rated health.

**Conclusions:**

Patients undergoing diagnostic evaluations for cancer based on non-specific symptoms experience a high prevalence of anxiety and affected quality of life prior to knowledge of the diagnosis. The predictive value of the baseline scores is important when assessing the psychological impact of undergoing diagnostic evaluations for cancer.

## Background

Undergoing diagnostic evaluations for suspected cancer can be experienced as frightening because of the threat of being seriously ill and because of the possible invasive investigations needed to confirm or refute malignant disease [1–5]. Previous studies investigating patient experiences in the diagnostic phase of cancer have primarily focused on patients diagnosed with cancer [3–7]. Few studies have examined the diagnostic experience of patients with an unknown diagnosis or with a non-cancer result. Although mostly limited to suspicions of breast cancer, these studies suggest that patients confronted with the possibility of a cancer diagnosis experience high levels of anxiety during the diagnostic phase [8–10]. A high level of anxiety can interfere with the ability to perform daily activities and receive necessary information and health care [2, 11, 12], and several studies have shown that health-related quality of life (HRQoL) might be affected [6–10]. Affected HRQoL in the diagnostic phase has been associated with increased anxiety and depression after diagnosis in patients diagnosed with cancer [11, 12]. The psychological impact of the diagnostic phase is additionally highlighted by studies of outcomes of breast cancer screenings, where patients with a non-cancer outcome might later experience psychological consequences [13, 14].

Danish studies have shown that approximately 50 % of all cancer patients initially present with vague symptoms or symptoms that are not specifically associated with cancer [13, 14]. A Cancer Patient Pathway (CPP) for patients with serious Non-Specific Symptoms and Signs of Cancer (NSSC-CPP) was therefore introduced in 2012 in Denmark. The objectives of this pathway are to offer patients an optimal evaluation and diagnosis, to minimize passive waiting time, and to improve quality of life during the diagnostic phase [15, 16]. A clinical coordinator is attached to the pathway to optimize logistics, and the aim is to diagnose or refute cancer or any serious illness within 22 days. According to the guidelines, patients are at referral to be informed about the suspicion of cancer [17].

The measurement of patient reported outcomes (PROs) prior to diagnosis might be crucial to provide a clear point of comparison for later measurements and could facilitate a more reliable interpretation of the results [18]. Moreover, understanding how patients cope with a potential cancer diagnosis is important in determining their HRQoL [18]. However, measuring coping with a life strain might be difficult. The concept of Sense of Coherence (SOC), although under discussion, has a broad theoretical base and a growing body of empirical evidence supporting its utility as a determinant for successful coping [18–22]. The concept of SOC is defined as an individual’s global view of life and as an inner resource for coping with stressful life events. The concept does not refer to a particular coping strategy [18]. SOC was originally believed to represent a staple dispositional orientation [19]. However, this degree of stability has been questioned by several studies [20–22], and it remains to be clarified if a stressful life experience, such as a cancer diagnosis, affects levels of SOC [22].

Patients referred to the NSSC-CPP are suspected of having cancer due non-specific or vague symptoms. Knowledge about how these patients experience the diagnostic phase and how this experience might be influenced by the final diagnosis can be used to enhance evidence based care in the diagnostic phase of cancer. Therefore, the aims of this study were as follows: 1) to assess HRQoL, symptoms of anxiety and depression, sense of coherence, and self-rated health as measures of PROs pre- and post-diagnosis in patients undergoing diagnostic evaluations for cancer; 2) to examine changes in PROs over time and in relation to the final diagnosis (cancer yes/no); and 3) to assess the predictive value of baseline scores, sense of coherence, self-rated health, socio-demographic factors, and co-morbidity on global QL, anxiety and depression after diagnosis.

## Methods

### Participants

A prospective survey study was carried out between October 1, 2013, and September 30, 2014, at four hospitals in the Capital Region, Denmark. All consecutive patients referred to the NSSC-CPP during the study period were eligible to participate in this study. The exclusion criteria were patients younger than 18 years of age, patients with cognitive disorders, and patients with language barriers or if referral to the NSSC-CPP was due to metastasis with unknown primary tumor. All participating patients were asked to complete the European Organisation for Research and Treatment of Cancer quality of life questionnaire (EORTC-QLQ-C30), the Hospital Anxiety and Depression Scales (HADS), the Sense of Coherence 13-scale (SOC-13) and Self-Rated Health (SRH) prior to diagnosis (baseline) and 30 days after referral (follow-up), a point in time when clinical evaluations should have been completed. The patients were aware of the suspicion of cancer prior to the baseline assessment. The follow-up questionnaire was sent with a stamped envelope. Patients who did not return the questionnaire were sent a reminder after 2 weeks.

### Questionnaires

HRQoL was assessed using the Danish version of the EORTC-QLQ-C30 questionnaire (version 3.0) [23]. The EORTC QLQ-C30 is a self-administered questionnaire that was developed to cover the multi-dimensional concept of HRQoL in cancer patients. The questionnaire consists of 30 items, which aggregate into one global health/QL scale, five functional scales (physical, emotional, role, cognitive and social functioning), three symptom scales (fatigue, pain and nausea/vomiting), and six single items assessing financial impact and various symptoms. Each item is answered on a four-point scale, from 1 (not at all) to 4 (very much), except for the global health/QL items. These items have seven response options ranging from 1 (very poor) to 7 (excellent) [23, 24]. The raw score of each scale/single item is linearly transformed according to the manual on a 0–100 scale [24]. A high score for the global health/QL scale and functioning scales represents a high level of quality of life and functioning. A high score for a symptom scale represents a high level of symptomatology. Missing items were imputed by the methods advocated by the EORTC QLQ research group [24]. Differences in mean scores of ten or more are regarded as clinically significant [25].

Anxiety and depression were assessed using the Danish version of the HADS. The HADS was developed to measure symptoms of anxiety and depression in somatically ill patients [26]. The scale is divided into an anxiety subscale (HADS-A) and a depression subscale (HADS-D), both containing seven intermingled items. The patients are asked to evaluate how they felt during the last week. Each item is scored between 0 and 3, where 0 is asymptomatic and 3 is considerable. The total score for each of the two subscales is between 0 (no sign of illness) and 21 (severe degree of anxiety or depression) [26, 27]. The HADS has been extensively used in the field of cancer [28], and several studies have shown good sensitivity and specificity with a cut-off score of 8+ for each of the two subscales [26, 29].

Sense of coherence was assessed using the 13-item version of Antonovsky’s SOC Scale (SOC-13). The SOC-13 contains three components: meaningfulness, comprehensibility, and manageability. All items are scored on a 7-point semantic differential scale. A total score is calculated, ranging from 13 to 91. A higher score indicates a better SOC [19]. The validity and reliability of this scale have been supported in numerous studies [30, 31], including cancer populations [32, 33].

The SRH was measured by two single items asking the participants to rate their overall current health and their current health compared to three months ago. Both items could be answered on a five point Likert scale ranging from excellent (5) to really poor (1). These single items have been shown to be a good predictor of morbidity and mortality regardless of the precise wording of the question [34–36].

At baseline and follow-up, participants were asked whether they completed the questionnaire before or after receiving knowledge of their diagnosis.

### Demographics and medical information

The demographic variables were collected at baseline and included self-reported age, gender, marital status, education and employment status. Information on clinical variables (symptoms at referral, duration of symptoms, exposures and smoking) was obtained from the patients’ medical records. Information on the diagnoses and co-morbidities, including previous cancer, was collected from the Danish National Patient Registry [37]. Co-morbidities were scored according to the Charlson’s Comorbidity Index [38].

### Ethics

All participants provided informed consent before data collection commenced. Approval from the National Committee on Health Research Ethics was not required (H-3-2013-061). The study was approved by The Danish Data Protection Agency (HIH-2013-034). The baseline characteristics for non-participants were obtained anonymously for dropout analyses.

### Statistical analysis

Categorical variables are described as counts (%), and continuous variables are described as means (SD) or medians with the 25 to 75th interquartile ranges (IQR). Differences in baseline characteristics were summarized and compared between patients diagnosed with cancer and patients who were not diagnosed with cancer using the Pearson’s χ2-test, Student’s unpaired T-test or the Wilcoxon rank sum test, as appropriate. The intra-group difference of the EORTC-QLQ-C30, HADS, SOC and SRH scores between baseline and follow-up within the diagnosis groups (cancer yes/no) were calculated using the Wilcoxon signed-rank test for continuous data and McNemar’s χ2-test for categorical data. The inter-group differences between the baseline and follow-up scores in the two diagnosis groups were calculated using the Pearson’s χ2-test for categorical data and the Wilcoxon rank sum test for continuous data, and the intra-group differences within the groups were estimated by the Wilcoxon rank sum test. The differences between the groups were also estimated by effect size and interpreted by Cohen’s criteria: <0.20 none, 0.20–0.49 small, 0.50–0.79 moderate and ≥0.80 large [39]. The analysis was restricted to subjects for whom both baseline and follow-up data were available.

The predictive value of the baseline values, socio-demographic factors, and co-morbidity on global QL was explored by bootstrapped multivariate linear regression with 2,000 repetitions. Logistic regression was used to estimate the predictive value of the included variables on clinical anxiety and depression (HADS score of ≥8). The baseline score, age, gender, cancer diagnosis (yes/no), duration of symptoms (weeks), previous cancer in patient, co-morbidities (0, 1, ≥2), marital status, education, occupation, time when the questionnaire was completed at follow-up (before or after diagnosis), and hospital site were included in all models in one single step. All tests were two-sided, with a significance level at *p* <0.05. The analyses were carried out using STATA 13.

## Results

### Patient characteristics

**Fig. 1:**
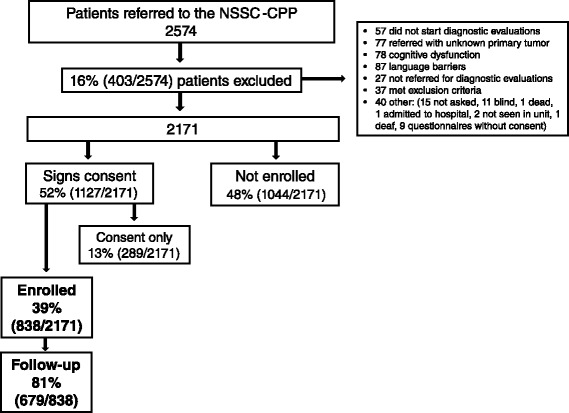
Flowchart of patient enrolment

A flowchart providing an overview of the participating patients is presented in Fig. 1. In total, 2,574 patients were referred to the NSSC-CPP at one of the participating hospitals during the study period; 403 (16 %) patients were initially excluded, and 1,044 (48 %) patients did not want to participate (‘Not enrolled’). Of the 1127 patients who provided informed consent, 289 (13 %) never completed the questionnaire (‘Consent only’), while 838 (39 %) returned a completed questionnaire and were enrolled in the study (‘Enrolled’). A total of 679 (81 %) participants completed the follow-up. There was no difference in the presence of a cancer diagnosis between the participants who completed the follow-up and those who did not complete the follow-up (*p*=0.4). Enrolled patients were more likely to be diagnosed with cancer than ‘consent only’ patients. Diagnosis was not available for the ‘not enrolled’ patients (Table 1). The baseline characteristics are presented in Table 2 for all enrolled patients and according to cancer diagnosis.

**Table 1:** Comparison between Enrolled, Consent only and Not Enrolled patients

	Enrolled	Consent only	Not enrolled	*p-value*
	838	289	1044	
Age, mean (SD), years	63.6 (13.5)	60.5 (17.1)	64.7 (16.4)	**<0.001** ^**a**^
Gender, *n* (%), women	443 (53)	162 (56)	591 (57)	0.25
Symptoms at referral, *n* (%)				
Weight loss	297 (35)	111 (38)	346 (33)	0.23
Pain	122 (15)	57 (20)	161 (15)	0.11
Suspicion of major illness/cancer	127 (15)	5 (2)	30 (3)	**<0.001** ^**c**^
Abnormal blood tests	106 (13)	36 (12)	118 (11)	0.63
Fatigue	105 (13)	40 (14)	151 (14)	0.48
Pathological lymph node	72 (9)	24 (8)	71 (7)	0.31
Anemia	71 (8)	28 (10)	74 (7)	0.28
Feeling ill	41 (5)	16 (6)	34 (3)	0.09
Night sweats	46 (5)	20 (7)	43 (4)	0.12
Loss of appetite/nausea	35 (4)	25 (9)	45 (4)	0.01^a^
Fever	34 (4)	10 (3)	34 (3)	0.65
Abdominal disorder	31 (4)	18 (6)	43 (4)	0.18
Increased contact to health system	2 (0.2)	1 (0.3)	0	0.48
Recurrent deep venous thrombosis	1 (0.1)	1 (0.3)	0	0.62
Increased use of medication	0	0	0	
Other	177 (21)	72 (25)	90 (9)	**<0.001** ^**b**^
Cancer diagnosis, *n* (%)	188 (22)	35 (12)		**<0.001** ^**a**^

^a^Significant difference between enrolled and consent only

^b^Significant difference between enrolled and not enrolled

^c^Significant difference between enrolled and consent only, and significant difference between enrolled and not enrolled

Significant results are higligthed in bold

**Table 2:** Baseline characteristics

		Cancer		
	All patients *n*=838	yes *n*=188	no *n*=650	*p*-value
Age, mean (SD), years	63.6 (13.5)	68.9 (10.5)	62.0 (13.8)	**<0.001**
Gender, n (%), men	395 (47)	107 (57)	288 (44)	**0.002**
Co-morbidity, n (%)				
0	393 (47)	12 (6)	381 (58)	
1	258 (31)	111 (59)	147 (23)	
≥2	187 (22)	65 (35)	122 (19)	**<0.001**
Duration of symptoms, weeks, median (IQR)	12 (6–26)	10 (4–20)	12 (8–26)	**<0.001**
Missing, n (%)	168 (20)	43 (23)	123 (19)	
Exposures, n (%)	158 (19)	29 (15)	129 (20)	0.18
Missing, n (%)	5 (0.6)	0	5 (1)	
Smoking, n (%)				
Never smoked	368 (44)	77 (41)	291 (45)	
Former smoker	221 (26)	60 (32)	161 (25)	
Smoker	208 (25)	39 (21)	169 (26)	
Missing, n (%)	41 (5)	12 (6)	29 (4)	0.11
Marital status, *n* (%)				
Married/co-inhabitant	565 (67)	120 (64)	445 (68)	
Separated/divorced	97 (12)	30 (16)	67 (10)	
Widow/widower	92 (11)	28 (15)	64 (10)	
Unmarried/single	80 (9)	9 (5)	71 (11)	
Missing, n (%)	4 (0.5)	1 (0.5)	3 (0.5)	**0.005**
Education, *n* (%)				
Compulsory <12 years	178 (21)	31 (16)	147 (23)	
Short <15 years/skilled worker	285 (34)	67 (36)	218 (34)	
Medium academic/trade	234 (28)	57 (30)	177 (27)	
Long academic/university level	131 (16)	32 (17)	99 (15)	
Missing, n (%)	10 (1)	1 (0.5)	9 (1)	0.37
Occupation, *n* (%)				
Employed	289 (35)	46 (25)	243 (37)	
Retired/disability pay	516 (62)	139 (74)	377 (58)	
Unemployed	29 (3)	3 (1)	26 (4)	
Missing, n (%)	4 (0.5)	0	4 (9.6)	**0.001**
Cancer in family, *n* (%)	162 (19)	36 (19)	126 (19)	
Missing, n (%)	20 (2)	4 (2)	16 (2)	0.96
Previous cancer in patient, n (%)	72 (9)	0	72 (11)	
Missing, n (%)	7 (1)	0	7 (1)	**<0.001**

Categorical variables are described as counts (%), and continuous variables are described as the mean (SD) or medians with the 25 to 75th interquartile ranges (IRQ). Differences between patients diagnosed with cancer and patients not diagnosed were estimated using the Pearson χ2-test or Wilcoxon rank sum test, as appropriate

Significant results are highligthed in bold

### Quality of life over time and in relation to the final diagnosis

The difference in the EORTC-QLQ-C30 scores over time in patients diagnosed and not diagnosed with cancer, in addition to the difference between the two diagnosis groups, are presented in Table 3.

**Table 3:** EORTC-QLQ-C30 scores at baseline and follow-up stratified by cancer diagnosis (yes/no), and the difference between diagnosis groups

	Cancer			Not cancer								
	Baseline	Follow-up	Difference within group^a^	Baseline	Follow-up	Difference within group^a^	Differences between groups at baseline^b^		Differences between groups at follow-up^b^		Difference between intra difference in groups^b^	
EORCT	*n*=125	*n*=125	*p-value*	*n*=549	*n*=549	*p-value*	*p-value*	*ES* ^c^	*p-value*	*ES* ^c^	*p-value*	*ES* ^c^
Global Health/QL, mean (SD)	53 (23)	56 (27)	0.19	53 (24)	60 (25)	**<0.001**	0.95	0.01	0.24	0.13	0.08	0.16
Functional scales, mean (SD)												
Physical functioning	74 (24)	70 (27)	0.42	75 (23)	76 (23)	0.39	0.78	0.06	0.07	0.25	0.28	0.28
Role functioning	57 (35)	56 (36)	0.64	63 (34)	68 (32)	**<0.001**	0.10	0.17	**<0.001**	0.38	0.06	0.22
Emotional functioning	74 (21)	73 (22)	0.39	70 (24)	75 (24)	**<0.001**	0.07	0.19	0.13	0.08	**<0.001**	0.35
Cognitive functioning	84 (21)	80 (23)	**<0.01**	79 (24)	79 (22)	0.35	**<0.01**	0.25	0.59	0.03	**0.001**	0.33
Social functioning	82 (26)	77 (30)	0.06	79 (26)	79 (28)	0.44	0.11	0.11	0.62	0.08	**0.03**	0.22
Symptom scales/items, mean (SD)												
Fatigue	45 (31)	44 (30)	0.86	46 (28)	40 (27)	**<0.001**	0.47	0.07	0.24	0.13	**0.003**	0.26
Nausea and vomiting	10 (17)	11 (19)	0.93	10 (17)	8 (16)	**<0.001**	0.75	0.01	0.08	0.18	0.08	0.20
Pain	31 (31)	30 (31)	0.33	35 (32)	32 (31)	**<0.01**	0.24	0.12	0.37	0.08	0.98	0.04
Dyspnea	27 (33)	25 (30)	0.52	22 (30)	19 (27)	**<0.01**	0.12	0.17	**0.02**	0.24	0.63	0.04
Insomnia	31 (34)	33 (33)	0.53	34 (35)	32 (33)	0.31	0.39	0.09	0.88	0.01	0.30	0.12
Appetite loss	33 (38)	32 (36)	0.92	29 (35)	23 (32)	**<0.001**	0.40	0.10	**0.02**	0.25	0.07	0.18
Constipation	15 (28)	19 (30)	0.47	15 (26)	14 (25)	0.32	0.59	0.01	0.12	0.18	0.26	0.19
Diarrhea	16 (27)	14 (26)	0.57	17 (28)	14 (24)	**<0.001**	0.65	0.04	0.64	0.01	0.36	0.06
Financial difficulties	5 (15)	9 (18)	**<0.01**	9 (24)	11 (25)	**0.04**	0.07	0.20	0.94	0.09	0.13	0.12

Bold values show statistical differences within and between groups

^a^Wilcoxon signed-rank test

^b^Wilcoxon rank sum test

^c^Effect size (ES) estimated by Cohen’s d; <0.20 = none, 0.20–0.49 = small, 0.50–0.79 = moderate, ≥0.80 = large

Patients diagnosed with cancer experienced overall less functioning and less or similar burden of symptoms at follow-up compared to baseline. The difference was statistically significant for cognitive function and financial difficulties. There was no significant difference in global QL between the two time points. Patients not diagnosed with cancer improved on all domains, except for cognitive and social functioning where there was no difference between the two time points. There was no difference between the groups at baseline, except for in cognitive function where patients diagnosed with cancer had a significantly higher score at baseline. Patients diagnosed with cancer had less role functioning at follow-up compared to patients with a non-cancer diagnosis; a finding that was both clinically and statistically significant. Patients diagnosed with cancer also experienced more dyspnea and appetite loss at follow-up compared to patients with a non-cancer diagnosis, and had a significantly larger decrease in emotional, cognitive and social functioning over time. Moreover, the change in the fatigue score between the two time points was significantly less than in patients not diagnosed with cancer. Overall, the effect sizes were small.

### Anxiety, depression, SOC and self-rated health over time and in relation to final diagnosis

The difference in the HADS anxiety and depression, SOC, and SRH scores over time and in relation to the final diagnosis are presented in Table 4.

**Table 4:** HADS, SOC and SRH scores at baseline and follow-up stratified by cancer diagnosis (yes/no), and the difference in change between diagnosis groups

		Cancer			Not cancer								
HADS	Range	Baseline	Follow-up	Difference within group^a^	Baseline	Follow-up	Difference within group^a^	Difference between groups at baseline^b^		Difference between groups at follow-up^b^		Difference between intra difference within groups^c^	
HADS Anxiety, n (%)		***n*** **=147**	***n*** **=147**	*p-value*	***n*** **=519**	***n*** **=519**	*p-value*	*p-value*	*ES* ^d^	*p-value*	*ES* ^d^	*p-value*	*ES* ^d^
Score <8		100 (68)	107 (73)		338 (65)	373 (72)							
Score ≥8		47 (32)	39 (27)		181 (35)	146 (28)							
Mean (SD)	0–21	5.6 (4.4)	5.4 (4.3)	0.58	6.3 (4.5)	5.4 (4.2)	**<0.001**	0.09	0.16	0.84	0.02	**0.01**	0.20
HADS Depression, n (%)		***n*** **=146**	***n*** **=146**		***n*** **=517**	***n*** **=517**							
Score <8		113 (77)	113 (77)		386 (75)	397 (77)							
Score ≥8		33 (23)	33 (23)		131 (25)	120 (23)							
Mean (SD)	0–21	4.4 (4.1)	4.5 (4.3)	1.00	5.0 (4.1)	4.5 (4.2)	0.25	0.45	0.07	0.90	0.01	0.07	0.20
Sense of Coherence		***n*** **=133**	***n*** **=133**		***n*** **=471**	***n*** **=471**							
SOC Total score, median (IQR)	13–91	76 (68–83)	75 (67–81)	0.07	73 (61–80)	73 (62–80)	0.07	**0.001**		**0.04**		0.42	
Self-rated Health													
Self-rated Health currently, n (%)		***n*** **=145**	***n*** **=145**		***n*** **=523**	***n*** **=523**							
(very) good		36 (25)	36 (25)	0.13	96 (18)	104 (20)	0.68	0.06		0.12			
fair		47 (32)	51 (35)		203 (39)	216 (41)		0.08		0.21			
(very) poor		62 (43)	58 (40)	1.00	224 (43)	203 (39)	0.23	0.49		0.85		0.22	
Self-rated Health compared to 3 months, n (%)		***n*** **=145**	***n*** **= 145**		***n*** **=522**	***n*** **=522**							
(very) good		10 (7)	24 (17)	0.12	44 (8)	149 (29)	**<0.001**	0.73		**<0.01**			
fair		54 (37)	60 (41)		248 (48)	264 (50)		**0.03**		0.06			
(very) poor		81 (57)	61 (42)	**0.02**	230 (44)	109 (21)	**<0.001**	**0.01**		**<0.001**		**<0.01**	

Bold values show statistical differences within and between groups

^a^McNemar’s χ2 for categorial data and Wilcoxon signed-rank test for continues data. For SRH the fair category was used as reference

^b^χ2 test for categorial data and Wilcoxon rank sum test for continues data

^c^Wilcoxon rank sum test of the mean intragroup difference

^d^Effect size (ES) estimated by Cohen’s d; <0.20 = none, 0.20–0.49 = small, 0.50–0.79 = moderate, ≥0.80 = large

There were no significant differences in the mean anxiety score between the groups at baseline or follow-up. Using the recommended cutoff score of eight points or more [26], 32 % of patients who were later diagnosed with cancer and 35 % of patients with a non-cancer diagnosis experienced clinical anxiety prior to knowledge of their diagnosis. The patients who were not diagnosed with cancer experienced a significantly larger decrease at follow-up than the patients with a non-cancer diagnosis.

The mean depression scores were lower than the anxiety mean scores, and 23 % of patients later diagnosed with cancer and 25 % of patients with a non-cancer diagnosis achieved a level of probable clinical depression at baseline. There were no significant differences between the groups at baseline or at follow-up, and there were no significant intra-group differences between the diagnosis groups. There were also no intra-group differences in the SOC-13 scores between baseline and follow-up. The patients diagnosed with cancer had a significantly higher SOC at baseline and at follow-up.

Forty-three percent of patients in both diagnosis groups rated their current health as poor or very poor at baseline, and both groups experienced an improvement after receiving knowledge of their diagnosis. There were no significant differences within or between the groups. At baseline, 57 % of patients diagnosed with cancer rated their health as poor or very poor compared to 3 months ago. Using the fair category as a reference, there was a significant improvement in SRH at follow-up compared to 3 months ago. A high proportion of patients who were not diagnosed with cancer also rated their health compared to 3 months ago as fair (48 %) or (very) poor (44 %) at baseline. There was a significant improvement at follow-up. There was a significant difference between the diagnosis groups at baseline and follow-up, and patients not diagnosed with cancer experienced a significantly larger increase in SRH compared to 3 months ago than patients with a non-cancer diagnosis.

### Predictors of quality of life, anxiety, and depression after a known diagnosis

The results from the regression analysis of predictors of global QL are presented in Table 5.

**Table 5:** Adjusted bootstrapet multiple linear regression analysis of the determinants of global quality of life at follow-up

	Global QL		
	Coeff	95 % CI	*p-value*
Intercept	29.90	(10.71; 49.09)	0.002
Baseline global QL	0.38	(0.28; 0.48)	**<0.001**
HADS Anxiety at baseline	0.08	(−0.42, 0.58)	0.75
HADS Depression at baseline	−1.19	(−1.81; −0.59)	**<0.001**
SOC at baseline	0.13	(−0.04; 0.30)	0.13
Self-rated health, currently at baseline			
(very) good	Reference		
fair	−3.13	(−7.28; 1.03)	0.14
(very) poor	−9.28	(−14.25; −4.32)	**<0.001**
Age	0.21	(0.04; 0.38)	**0.02**
Co-morbidity			
0	Reference		
1	−5.14	(−8.63; −1.66)	**0.004**
≥2	−6.28	(−10.56; −2.01)	**0.004**
Occupation			
Employed	Reference		
Unemployed	−8.32	(−16.16; −0.49)	**0.04**
Retired/disability/early retirement	−3.97	(−8.14; 0.19)	0.06

Non-significant predictors including gender, time of follow-up completion, SRH compared to 3 months ago at baseline, cancer diagnosis (yes/no), marital status, education, previous cancer in patient and hospital were excluded from the table. *R* ^2^ = 0.48

Significant results are highligthed in bold

The strongest predictors of global QL after a known diagnosis were the baseline global QL score, baseline depression score, (very) poor current SRH at baseline, age, co-morbidity and unemployment. None of the included variables reached a score difference of ten, and therefore, none of the variables had a clinically significant impact.

The results of the regression analyses of anxiety and depression after a known diagnosis are presented in Table 6.

**Table 6:** Adjusted logistic regression analysis of the determinants of clinical anxiety and depression at follow-up

	Anxiety			Depression		
	OR	95 % CI	*p-value*	OR	95 % CI	*p-value*
Baseline global QL	0.98	(0.96; 0.99)	**0.01**	0.99	(0.97; 1.00)	0.06
HADS Anxiety at baseline						
Score <8	Reference			Reference		
Score ≥8	9.80	(5.79; 16.60)	**<0.001**	1.63	(0.92; 2.89)	0.09
HADS Depression at baseline						
Score <8	Reference			Reference		
Score ≥8	1.31	(0.71; 2.43)	0.39	8.70	(4.70; 16.10)	**<0.001**
SOC at baseline	0.96	(0.93; 0.98)	**<0.001**	0.96	(0.94; 0.99)	**0.005**
Self-rated health, currently at baseline						
(very) good	Reference			Reference		
fair	1.16	(0.53; 2.54)	0.71	2.31	(0.88; 6.07)	0.09
(very) poor	1.29	(0.59; 2.85)	0.51	2.66	(1.04; 6.78)	**0.04**
Co-morbidity						
0	Reference			Reference		
1	1.38	(0.74; 2.58)	0.31	1.75	(0.88; 3.46)	0.11
≥2	0.85	(0.42; 1.71)	0.65	2.17	(1.06; 4.45)	**0.03**
Follow-up post diagnosis	0.49	(0.27; 0.88)	**0.02**	1.02	(0.54; 1.96)	0.94

Non-significant predictors including age, gender, SRH compared to 3 months ago at baseline, cancer diagnosis (yes/no), marital status, education, occupation, previous cancer in patient and hospital were excluded from the table. McFadden’s *R* ^2^ = 0.37 for anxiety model and 0.38 for depression model. Hoswer and Leweshow test was 0.56 for both models

Significant results are highligthed in bold

Anxiety at baseline was found to be the most significant predictor of clinical anxiety after a known diagnosis, along with the degree of SOC at baseline, the baseline global QL score, and follow-up completed post-diagnosis. The strongest predictors of clinical depression after a known diagnosis were depression at baseline, degree of SOC at baseline, (very) poor current SRH at baseline and having two or more co-morbidities.

## Discussion

### Main findings

In this prospective study, a broad spectrum of PROs from patients undergoing diagnostic evaluations for cancer because of non-specific symptoms was assessed in relation to changes over time and the final diagnosis. Patients suspected of cancer have similar HRQoL scores at the beginning of diagnostic evaluations irrespective of their final diagnosis. Goossens-Laan [40] found similar results in their study on pre-diagnostic HRQoL in patients suspected of having bladder cancer. Patients with a non-cancer diagnosis improved in global QL, role and emotional functioning, and they experienced fewer symptoms after a known diagnosis. Limited improvement over time was seen in patients diagnosed with cancer. The patients diagnosed with cancer were most likely early in their cancer trajectory at the time of follow-up and therefore were still affected by the novelty and insecurity of the situation.

Cognitive functioning was significantly higher at baseline and deteriorated significantly more over time in patients diagnosed with cancer. Cognitive function is characterized by items asking how current illness might affect concentration and memory. Although the baseline score among cancer patients was higher than the other functional domains, it is similar or lower than scores from both normative and cancer reference groups [41]. Moreover, patients diagnosed with cancer had significantly higher SOC scores than patients with a non-cancer diagnosis and a high SOC might serve as a determinant for successful adaption to stressful situations [19].

Patients diagnosed with cancer also experienced more dyspnea and appetite loss after a known diagnosis compared with the non-cancer patients. Initiation of cancer treatment could have an impact on these symptoms. However, although we did not have information on initiation of treatment, it is likely that few, if any, cancer patients had started treatment at the time of follow-up assessment, because of the short time period between referral and follow-up.

The most important impairment areas over time for patients diagnosed with cancer compared to patients with a non-cancer diagnosis were emotional, cognitive and social functioning, and fatigue. These areas have previously been identified as areas of concern in studies with specific cancer patients prior to and after receiving knowledge of their diagnosis [6, 7, 18, 42]. Affected role functioning appears to be transient, whereas emotional functioning and fatigue might continue for a long period after the initial diagnosis and treatment [42].

Nearly one-third of patients experienced clinical anxiety at baseline irrespective of the final diagnosis. Several studies have reported a prevalence of anxiety ranging between 46 % and 73 % in the diagnostic phase of breast or lung cancer [12, 43–46]. These studies included patients suspected of having a specific type of cancer due to radiological or symptomatic findings, thereby making the possible threat of illness more specific. Patients referred to the NSSC-CPP are not suspected of a specific cancer at referral. The most common symptoms at referral were non-specific in nature, which could explain the lower levels of anxiety in our study. Similar to our results, previous research has suggested that patients who receive a non-cancer diagnosis experience a larger decrease in anxiety levels after known diagnosis than patients diagnosed with cancer [2, 9, 10, 43, 45, 46]. However, it is important to highlight that 28 % of patients in the non-cancer group were experiencing clinical anxiety at follow-up. Receiving a non-cancer diagnosis can have negative psychological effects and possibly delayed cancer diagnosis in case of subsequent cancer symptoms [1, 47, 48]. Thus, it is essential to acknowledge the possible unintended consequences of a non-cancer result, and provide sufficient information and support to patients receiving a non-cancer diagnosis [48].

The strongest predictors of global QL after diagnosis were the baseline global QL and depression scores, (very) poor current SRH at baseline, age, co-morbidity and unemployment. Age, co-morbidity and unemployment have been associated with poorer quality of life in population studies [49–51]. Baseline HRQoL has been shown to be predictive of health outcomes, such as the survival rate and response to treatment, in cancer patients over time [52]. The global QL domain and SRH items are similar in wording, which could explain the strong association seen in the analysis [34–36]. Baseline anxiety and depression have been significantly associated with global QL impairment regardless of patients’ demographic and clinical characteristics [12]. Moreover, baseline anxiety and depression scores were also associated with clinical anxiety and depression after diagnosis. Our results therefore highlight the importance of including pre-diagnostic baseline assessments when looking at the psychological impact of a cancer diagnosis [53].

The degree of SOC was a significant predictor of clinical anxiety and depression after diagnosis; a finding supported by several studies [32, 54]. A high SOC has been associated with better perceived health and quality of life within different samples, including cancer patients [32, 55, 56]. SOC did not change over time, thereby supporting the original perception of SOC as a staple dispositional orientation with limited fluctuations in stressful situations [19].

Receiving a cancer diagnosis did not have an effect on global QL, anxiety or depression scores after known diagnosis. The risk of anxiety increased by nearly 50 % in patients who completed the follow-up prior to receiving knowledge of their diagnosis, highlighting the impact of the diagnostic phase irrespective of final diagnosis. Knowledge of diagnosis might be protective of clinical anxiety, and several studies have shown that the timeliness of diagnosis might have a beneficial effect on anxiety and depression irrespective of the final diagnosis [4, 9, 43].

### Strengths and limitations of the study

A major strength of this study is the prospective, multicenter design with a large number of consecutive patients. Diagnosis and co-morbidity data were collected via the National Patient Registry, which is considered to be precise and valid [37]. Patients were encouraged to complete the baseline questionnaire prior to receiving knowledge of their diagnosis. However, as patients were experiencing symptoms and were informed about the suspicion of cancer at baseline, this questionnaire might not render a true baseline measurement. Overall, the effect sizes were low and clinical differences in scores were found in the role functioning domain only. Thus, the clinical impact of the observed difference within and between the diagnosis groups may be limited. However, this could be because the follow-up was collected too close to the diagnostic phase to assess the impact of diagnosis in patients suspected of cancer. The focus of this study was to assess patient experience from referral to diagnosis, as this has not previously been described in this patient population. Although of interest, a long-term follow-up goes beyond the scope of the study.

The overall response rate was low; only 39 % of eligible patients participated. However, a low response rate does not necessarily indicate non-response bias [57, 58] because the differences between enrolled patients and non-participating patients were small. ‘Consent only’ patients were younger and less likely to have cancer, and a possible explanation for their decision not to enroll might be that they were eager to return to normal life. Enrolled patients were more often referred to the NSSC-CPP due to suspicion of having cancer or other reasons, indicating that the data collection methods were not precise enough. A recent study found that patients referred to the NSSC-CPP consist of a very heterogeneous group presenting with over 80 different symptoms [59]. Similar to other studies, we found weight loss, pain and fatigue to be the most common symptoms at referral [59]. We found an overall probability of cancer of 22 % in our sample, which is higher than in other studies [59, 60], but similar to the national surveillance data [17]. Because only four and 11 patients had a prior history of anxiety or depression, respectively, a prior history of anxiety and/or depression was not included in the multivariate analysis due to the small numbers.

## Conclusion

Patients suspected of having a cancer illness experience a high prevalence of anxiety and had an affected quality of life prior to receiving knowledge of their diagnosis. Patients who were not diagnosed with cancer experience a large improvement compared to patients diagnosed with cancer. The predictive value of the baseline PRO scores are important when assessing the psychological impact of undergoing diagnostic evaluations for cancer. Further research is needed to explore any long-term psychological implications of going through diagnostic evaluations for possible cancer.
